# Medical Association of Belgium

**Published:** 1836-04

**Authors:** 


### MEDICAL ASSOCIATION OF BELGIUM.

The first meeting of this new Association was held at Brussels on the 24th of September, 1835, and the six following days. The October number of the *Bulletin Médical Belge* is wholly occupied with an account of the proceedings.

September 24th. Dr. Marinus commenced the business by an oration. He stated the objects of the Association to be the advancement of medical science and union amongst medical men, and quoted the following passage, which pleasantly contrasts these assemblies, so characteristic of the present day, with those of the middle ages. "In the middle ages (says M. Poujoulat,) they assembled to discuss theological questions, to establish doubtful points of Christian faith; red hats and bishops' mitres were gathered from all quarters into one spot. Others met to cut and hack each other beneath the auspices of noble ladies, or to settle the affairs of love; and barons, minstrels, and knights, poured in from all sides: but the day for councils, tournaments, and courts of love, is past, and Associations for Science have replaced all the pompous spectacles of the Church and of Chivalry." He traced the history of the annual Scientific Meetings, from the first which was held at Leipsic in 1822, owing to a suggestion of Professor Ocken, and explained the manner in which they promoted science. The examples of Vesalius, Van-Helmont, and other Belgians whose names are distinguished in the annals of medicine, were urged as reasons why Belgium should not be behind-hand in the march of improvement. The speaker regretted the prejudice existing against native authors, which is so great that a Belgian in order to obtain readers has printed his works in Paris; and he entreated that the new medical journal now published in Brussels might be supported by native talent. This address was ordered to be printed.

September 25th. Dr. Fromont opened the meeting by an address in favour of the Association. M. Marcq followed, and gave a sketch of the present state of medical opinion: as an ardent disciple of Broussais he condemned the more modern eclecticism. "Water," says he, "is my universal panacea, it is the only remedy in which I rely, and not without success, in the adynamia of typhus and in the cold stage of cholera." He declaimed against homœopathy, and concluded by moving a resolution expressive of the disbelief of the Association in this new doctrine. This was negatived, as in the discussion which followed a few of the members expressed their adhesion to the doctrines of Hahnemann.

M. Burggraeve described and showed drawings of some human monsters.

September 26th. The discussion was resumed on homœopathy.

M. Bosch read two cases and observations upon them. The first was that of an infant uttering cries in utero. A woman had been in labour twenty-four hours when M. Bosch was called in. Six hours previously the waters had been discharged, and the midwife, to hasten delivery, had constantly introduced her hand into the vagina, and perhaps into the uterus, notwithstanding the cries of the patient. The parts were swollen and painful: the position of the head was transverse, the turn into the sacrum not having commenced. Whilst applying the forceps M. Bosch heard the cry of the fœtus, and on expressing his surprise, the father and five attendants said they had heard the same noise shortly after the waters came away. The cry was repeatedly heard before the delivery was effected. The child died twenty-four hours after birth. There are two cases on record of the same nature, and in a medico-legal point of view it is important. Orfila admits that a child may cry in utero if its mouth is at the os uteri: here however this was not the case. The introduction of the hand might have been the means of introducing air into the uterus. In drawing any conclusions from the hydrostatic test, the possibility of such a case as this should be borne in mind.—*2d Case.* M. Bosch examined a woman at the ninth month of pregnancy, the superior aperture of whose pelvis in the antero-posterior diameter was contracted to two inches and three quarters, from rachitis. He was on the point of prognosticating the impossibility of delivery and the necessity of the Cæsarian section, or of the division of the symphisis pubis, when on examining the os uteri he found that the head of the child had been developed in the cavity of the pelvis, which it filled: the inferior aperture was not contracted. The labour was easy and natural. This circumstance shows the possibility of natural delivery in a woman with so deformed a pelvis that the Cæsarian operation might have been required on a previous occasion.

M. Fallot proposed that the legislature be petitioned to determine expressly by law the question of medical responsibility. Carried.—M. Jullien proposed that a board of health be established at Brussels, and in the chief towns, as in Paris, to act in concert with the municipal body in taking measures for the public health. Another request was also made and carried, that the government should adopt the necessary measures for the institution of an Academy of Medicine.

September 28th. Discussion on M. Bosch's case.

September 29th. M. Branders detailed the particulars of a case of intermittent mental derangement of a man forty-five years old: the intermittence, which is of the tertian type, has lasted many years. During the paroxysms he is very violent, and would injure himself were he not confined with the strait-waistcoat and his head guarded with pads. He is so well aware, in his lucid intervals, of his deranged propensities, that he submits willingly to these precautions, and allows that they save his life. To be printed.

M. Marinus proposed that the government be petitioned to suppress the laws concerning patent medicines; and to expedite the publication of the Belgian Pharmacopœia. Carried.

September 30th. M. Valerius spoke at some length on the necessity of adopting means for raising the standard of "pharmaciens" in Belgium, who as a body are ignorant and deficiently educated. He confined himself to general statements, and the subject was deferred until the next session. A discussion followed on the selection of a subject for a prize of one thousand francs, which sum had been offered by a friend of medical science for the best essay on any subject that the Belgian Association should consider most useful to humanity. The following was ultimately fixed upon. "To set forth and determine the medical means and administrative regulations best adapted to put a stop to, or diminish, the propagation of syphilis." Essays legibly written in Latin, French, English, or German, to be directed (carriage paid) to Dr. Dieudonné, secrétaire de la commission permanente, rue de l'Empereur, No. 29. Each essay to be headed by a motto, and accompanied by a sealed letter directed with the same motto, and containing the names, titles, and abode of the writer. This concluded the business of the Association. Seventy-two members were present; ten others, who were unable to attend, enrolled their names.

*Bulletin Médical Belge, No.* 10, *Octobre*, 1835.

